# The Change of Soluble Programmed Cell Death-Ligand 1 in Glioma Patients Receiving Radiotherapy and Its Impact on Clinical Outcomes

**DOI:** 10.3389/fimmu.2020.580335

**Published:** 2020-10-30

**Authors:** Xing-Chen Ding, Liang-Liang Wang, Yu-Fang Zhu, Yan-Dong Li, Shu-Lun Nie, Jia Yang, Hua Liang, Ralph R. Weichselbaum, Jin-Ming Yu, Man Hu

**Affiliations:** ^1^Department of Oncology, Shandong First Medical University and Shandong Academy of Medical Sciences, Jinan, China; ^2^Department of Radiation Oncology, Shandong Cancer Hospital and Institute, Shandong First Medical University and Shandong Academy of Medical Sciences, Jinan, China; ^3^Department of Radiation and Cellular Oncology, Ludwig Center for Metastasis Research, The University of Chicago, Chicago, IL, United States; ^4^Department of Anesthesiology, Affiliated Hospital of Jining Medical University, Jinan, China

**Keywords:** soluble programmed cell death-ligand 1, glioma, radiotherapy, clinical significance, prognosis

## Abstract

**Background:**

The programmed cell death ligand 1 (PD-L1) plays a key role in glioma development. However, due to the specificity of glioma’s anatomical position, the role of its expression as a tumor biomarker is limited. It has been proven that the levels of soluble programmed death-ligand 1 (sPD-L1) are associated with prognosis in many malignancies including glioma. However, the expression of sPD-L1 in glioma patients receiving radiotherapy (RT) remains unclear. The purpose of this study was to evaluate the concentration of sPD-L1 in the plasma of glioma patients before and after RT and to explore its relationship with clinical outcomes.

**Methods:**

Between October 2017 and September 2018, glioma patients treated with RT (30 ± 10 Gy, 2 Gy/f) were enrolled, and blood samples were collected before and after RT. We quantified the sPD-L1 levels by enzyme-linked immunosorbent assay (ELISA). The isocitrate dehydrogenase-1 (IDH-1) mutational status and Ki-67 expression of tumors were evaluated by immunohistochemistry. Glioma murine model were used to address whether circulating sPD-L1 molecules are directly targeted by an anti-PD-L1 antibody. The associations between sPD-L1 and clinical features were assessed with Pearson’s or Spearman’s correlation analysis. The progression-free survival (PFS) and overall survival (OS) were determined by the Kaplan-Meier method.

**Results:**

Sixty glioma patients were included, with a median age of 52 years. The proportions of grade I, II, III, and IV gliomas were 6.7%, 23.3%, 28.4%, and 41.6%, respectively. The baseline sPD-L1 levels were significantly associated with tumor grade, IDH-1 mutation status and Ki-67 expression. Using 14.35 pg/ml as the cutoff, significantly worse PFS and OS were both observed in patients with higher baseline levels of sPD-L1 (*P* = 0.027 and 0.008, respectively). RT significantly increased the mean level of sPD-L1 (*P* < 0.001). Further analysis showed that the level of sPD-L1 in IDH-1 mutation patients was higher than that in wild-type patients. Furthermore, an analysis of glioma murine model indicated that anti-PD-L1 antibody combine with RT can be a potentially powerful cancer therapy.

**Conclusion:**

This study reported that sPD-L1 might be a potential biomarker to predict the outcome in glioma patients receiving RT. The elevated level of sPD-L1 after RT suggested that the strategy of a combination of immune checkpoint inhibitors and RT might be promising for glioma patients, especially for those with IDH-1 mutations.

## Introduction

Gliomas are the most common primary brain tumors, with a 5-year overall survival (OS) of approximately 36% (1). Glioblastoma (GBM) with only 5.6% of 5-year OS, is the most aggressive form making up 54% of all gliomas (1). Despite neurosurgical resection and adjuvant radio- and chemotherapy prolonging patient survival times, most glioma patients relapse and have limited their life expectancy. The reasons for the failure of conventional therapies include the protection of tumor cells by the blood–brain barrier, as well as invasive tumor growth in an essential organ, which limits the utility of local therapy (2). It is anticipated that novel effective approaches will be urgently required for the systemic treatments of gliomas.

Anti- programmed death 1/programmed death ligand 1 (PD-1/PD-L1) immunotherapy has shown clinical efficacy against many different solid tumor types and hematological malignancies (3–6). However, clinical trials of anti-PD-1/PD-L1 immunotherapy for glioma are relatively delayed (7, 8). Only one phase III clinical trial, Checkmate 143, has been completed; however, nivolumab did not exhibit increased survival benefits compared with bevacizumab (9). It seems that the PD-1/PD-L1 axis only plays one role in the malignant biological behavior of gliomas, while other molecular signaling networks may also play indispensable roles. Radiation is commonly used to treat glioma patients and has been identified to activate immune responses (10–12). Thus, researchers tried to explore the clinical efficacy of immunotherapy (nivolumab) + radiotherapy (RT) ± temozolomide (TMZ) in newly diagnosed glioblastoma patients in some ongoing phase III clinical trials, including Checkmate 498 (NCT02617589) and Checkmate 548 (NCT02667587). However, challenges remain to be addressed to maximize the efficacy of this promising combination. One of these challenges is the identification of biomarkers to assess the dynamic changes in the immune system at the level of patients undergoing RT.

To date, a number of candidate biomarkers of the immune response during and after RT, including both circulating and cellular, have been reported (13–17). Increasing evidence suggests that the expression of soluble PD-L1 (sPD-L1) in the blood is significantly associated with prognosis in gliomas and several extracranial malignancies (18–20). The elevated circulating and cerebrospinal fluid sPD-L1 levels were associated with aggressive biological activities in glioma patients (21). Similarly, high sPD-L1 levels were found which were correlated with abdominal organ metastasis in patients with advanced non-small cell lung cancer (NSCLC) (18) and increased mortality risk in patients with hepatocellular carcinoma (HCC) (20). Moreover, both sPD-1 and sPD-L1 could also acted as useful biomarkers to predict the outcome of PD-1 inhibition therapy in melanoma patients (22). These preliminary results prompted us to further investigate the application of sPD-L1 in the treatment of tumors.

Given the limited evidence that sPD-L1 may be a biomarker to predict the response to immunotherapy in gliomas, our study was performed to evaluate plasma concentrations of sPD-L1 before and after RT in glioma patients and to investigate the relationship of sPD-L1 levels with clinical outcomes. We hypothesized that circulating sPD-L1 molecules in the blood would deliver systemic inhibitory messages that could globally adversely impact antitumoral immune responses.

## Materials and Methods

### Patients

In this study, 60 patients were recruited. All of them were diagnosed primary or recurrent glioma, then treated with RT for glioma in Shandong Cancer Hospital between October 2017 and September 2018. The study protocol was approved by the Ethics Committee of the Shandong Cancer Hospital, and all patients gave their written informed consent prior to study inclusion. The optimal treatment option was determined by a multidisciplinary tumor board in accordance with our institution’s treatment policy. RT was performed using conventional fractionated RT. It was considered in patients on an individual basis, with a total dose of 54–60 Gy in 30 fractions (f). Concurrent peroral chemotherapy with TMZ was administered at 75 mg/m^2^ daily during RT. Bevacizumab combined with CCRT was administered at 10 mg/kg (repeated every 2 weeks). If the patient was required surgery first, RT was initiated within 8 weeks after surgery. After the scheduled treatment was finished, regular follow-up was conducted every 3 months with imaging studies and tumor marker analyses.

### Blood Sampling

Blood (5–10 ml) was collected from the patients before RT (0 Gy) and after RT (30 ± 10 Gy, 2 Gy/f) using aseptic tubes containing EDTA (5 ml). The blood samples were centrifuged at 2000 rpm for 10 min at 4°C to separate the plasma. Additional centrifugation for 10 min was performed to produce cell-free plasma, after which the plasma aliquots were immediately frozen at -80°C for further analysis.

### Soluble PD-L1 Measurement

Patients’ sPD-L1 levels were measured using the human PD-L1 simple-step enzyme-linked immunosorbent assay kit (ab214565, Abcam, USA). In brief, 50 μl of standards at different concentrations and patient plasma samples were added to the wells. Subsequently, 50 μl of PD-L1-conjugated antibody was added, incubated for 1 h at room temperature and then washed 3 times. Next, the substrate solution was applied for the color reaction, which was stopped with stop solution, and the absorbance was immediately measured at 450 nm using an enzyme-linked immunosorbent assay reader (VERSA max microplate reader; USA). The sPD-L1 level was calculated according to standard curves. The minimum detectable concentration of sPD-L1 was 2.91 pg/ml.

### Immunohistochemistry and Molecular Pathology

The immunohistochemistry (IHC) sample slides were reviewed by two neuropathologists, and a systematic neuropathological review was based on the 2007 WHO classification of CNS tumors. Tumor tissue was formalin fixed and paraffin embedded according to standard laboratory practice. Specimens were stained with antibodies against isocitrate dehydrogenase-1 (IDH-1) R132H (clone H09, 1:50 dilution; Maxim, China) and Ki-67 (MIB1; Santa Cruz, Shanghai, China, 1:50 dilution). Cells with pale brown granular deposits were considered to have IDH-1 mutational status and be Ki-67 positive. The Ki-67 index is the percentage of positive cells in the densest visual field. IHC analyses were then performed with a quantitative approach under a light microscope.

### Glioma Murine Model

C57BL/6 mice (6–8 weeks) were maintained in SPF laboratory conditions and were subcutaneously injected with 2 x 10^6^ cells (GL261 cells) in the right flank (day 0). When the tumor reached a volume of approximately 100 mm^3^ (approximately 10 days), tumors received one dose of 20 Gy ionizing radiation (IR), and/or 200 μg of anti-PD-L1 antibody (clone 10F.9G2) or isotype control antibody. The sPD-L1 level in the plasma was measured using the mouse PD-L1 DuoSet ELISA (DY1019-05, R&D Systems, USA) before and after IR. Tumor volume was measured twice weekly with calipers, and tumor volume was approximated using the equation for an ellipsoid: (width)^2^ × length/2. Mice were sacrificed when tumors reached 2,000 mm^3^.

For the suppression assay, CD8^+^ T cells were purified from mouse lymph nodes (inguinal, axillary, brachial, superficial cervical, and lumbar) and spleens by CD8 isolation kit (Stemcell, Vancouver, BC). 2 × 10^5^ of naïve CD8^+^ T cells and the plasma of mice after IR were co-cultured in complete RPMI1640 with the presence of 100 μM of β-mercapitoethanol (Sigma) and 2 μg/ml αCD28 (Biolegend, clone 37.51) in the wells of a flat-bottom 96-well plate coated with 5 μg/ml αCD3 (Biolegend, clone 145-2C11). Cells were harvested after 3 days, stained for CD8^+^ T cells (Biolegend, clone 53-6.7) and analyzed by flow cytometry.

All experiments related to animals were strictly performed in accordance with guidelines approved by the Ethics Committee of Shandong Cancer Hospital.

### Statistical Analysis

Continuous variables are shown as the mean ± the standard deviation (SD) and the minimum-maximum range. The differences between the two groups were calculated using the t-test or the Mann–Whitney U-test according to the normality of the data. The Kruskal–Wallis test was used for the comparison of three or more groups. The *post hoc* Bonferroni test was used for multiple comparisons. Correlations between the sPD-L1 level and clinical factors were analyzed using Pearson’s correlation analysis or Spearman’s correlation analysis for continuous variables. The chi-squared test or Fisher’s exact test were used for categorical variables. Receiver operating characteristic (ROC) curve analysis was used to determine the optimal cut-off value of sPD-L1 and Ki-67 expression rates. The survival duration was calculated from the date of disease diagnosis (RT start) to the corresponding event. The Kaplan-Meier method with the log-rank test was used to compare survival between groups. Multivariable analysis was carried out by the Cox regression hazard model. The dynamics of sPD-L1 in the plasma were analyzed by the mixed-model approach. All statistical tests were two-sided, and *P* values <0.05 were considered to be significant. All data were analyzed using IBM SPSS software version 22.0 (IBM, New York, USA). Figures were made by GraphPad Prism version 5.00 (San Diego, California, USA).

## Results

### Patient Characteristics and Survival Outcome

In this study, 60 glioma patients who had measurable tumors and received RT in Shandong Cancer Hospital were enrolled. Of them, 33 were female and 27 were male, with a median age of 52 years (range, 18–75). Fifty-two out of 60 patients received a pathological diagnosis (20 *via* subtotal resections and 32 *via* tumor biopsies), and the other eight patients were diagnosed with GBM by radiological findings based on the current guidelines. Twenty-five patients (41.6%) had pathological grade IV gliomas, 17 patients (28.4%) had grade III gliomas, 14 patients (23.3%) had grade II gliomas, and four patients (6.7%) had grade I gliomas. Of the 60 patients, 42 (70%) patients received RT plus TMZ (CRT), 10 (16.7%) patients received RT plus both TMZ and bevacizumab (CRT+T), and the other eight patients received only RT. The clinical baseline characteristics and outcomes of 60 glioma patients were systematically reviewed, and the results are summarized in **Table 1**.

**Table 1:** Patient characteristics and outcomes.

Parameter	Patients
Epidemiology	
Patients, n	60
Gender, m/f (%)	27/33 (45/55)
Age, median, range	52, 18–75
Karnofsky performance score, median, range	90, 60–90
Numerical Rating Scale (pain measurement), median, range	0, 0–6
Nutritional Risk Screening 2002, median, range	1, 0–3
Pathological grading	
I n (%)	4 (6.7)
II n (%)	14 (23.3)
III n (%)	17 (28.4)
IV n (%)	25 (41.6)
Tumor position	
Left brain, n (%)	27 (45)
Right brain, n (%)	11 (18.3)
Brain stem, n (%)	9 (15)
Other, n (%)	13 (21.7)
IDH-1 status	
Mutation, n (%)	15 (25)
Wild type, n (%)	25 (41.7)
Unknown, n (%)	20 (33.3)
Ki-67 expression (cut off rate)	
≤27.5%, n (%)	28 (46.6)
>27.5%, n (%)	16 (26.7)
Unknown, n (%)	16 (26.7)
Diagnostic style	
Subtotal resection, n (%)	20 (33.3)
Tumor biopsy, n (%)	32 (53.4)
No biopsy, n (%)	8 (13.3)
Type of therapy	
Radiotherapy + TMZ, n (%)	42 (70)
Radiotherapy + TMZ + bevacizumab, n (%)	10 (16.7)
Radiotherapy only, n (%)	8 (13.3)
Follow-up time, median, range	28.7 (5.4–38.7)
Recurrence and/or metastasis at last follow-up	
Yes, n (%)	23 (38.3)
No, n (%)	28 (46.7)
Unknown, n (%)	9 (15)
Alive at last follow-up	
Yes, n (%)	38 (63.3)
No, n (%)	22 (36.7)
Unknown, n (%)	0 (0)

### Association Between Baseline sPD-L1 Levels and Clinical Factors

**Figure 1:**
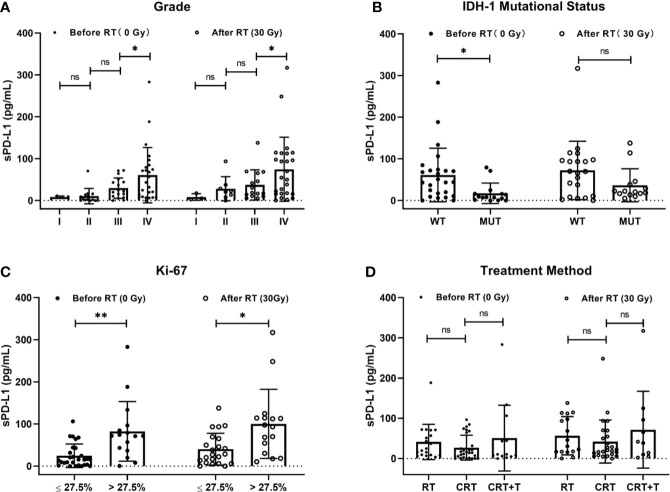
Correlations of soluble PD-L1 (sPD-L1) levels with clinical factors. **(A)** Grade (I, II, III, and IV), **(B)** IDH-1 mutational status (mutation and wide type), **(C)** Ki-67 (≤27.5% and >27.5%), and **(D)** treatment method (Radiotherapy (RT), RT plus chemotherapy (CRT), CRT and beacizumab (CRT+T) before and after RT. ns, non-significant. *P < 0.05, **P < 0.01.

To investigate the association between baseline sPD-L1 and clinical factors, we measured the sPD-L1 levels in the plasma of 60 patients before radiation therapy and detected the IDH-1 mutational status of 40 patients and the expression of Ki-67 in 44 patients by IHC. The mean level of baseline sPD-L1 was 47.39 ± 59.01 pg/ml (range, 0 - 283.13 pg/ml). Spearman correlation analysis showed that the baseline sPD-L1 level was positively associated with tumor grade (*r* = 0.495, *P* < 0.001), IDH-1 mutational status (*r* = 0.379, *P* = 0.016), and Ki-67 expression rate (*r* = 0.434, *P* = 0.003). With the increase in glioma stage, the mean level of baseline sPD-L1 tended to increase (stage I: 8.18 ± 2.70 pg/ml; stage II: 10.52 ± 18.35 pg/ml; stage III: 29.65 ± 24.23 pg/ml, and stage IV: 60.60 ± 65.95 pg/ml, **Figure 1A**). Compared to patients with IDH-1 wild-type (WT) tumors, patients with IDH-1 mutation (MUT) tumors showed markedly lower levels of baseline sPD-L1 in plasma (17.28 ± 24.59 pg/ml *vs.* 61.18 ± 64.30 pg/ml, **Figure 1B**). In addition, we found that the sPD-L1 level was higher in patients with Ki-67 > 27.5% than in those with Ki-67 ≤ 27.5% (82.58 ± 70.77 pg/ml *vs.* 24.68 ± 27.89 pg/ml, **Figure 1C**). As expected, there were no significant associations between sPD-L1 levels and other factors, *e.g.*, sex, age, Karnofsky Performance Status (KPS) score, Numerical Rating Scale (NRS), Nutritional Risk Screening 2002 (NRS 2002), or tumor location.

### Correlation Between Baseline sPD-L1 Levels and Clinical Outcomes

**Figure 2:**
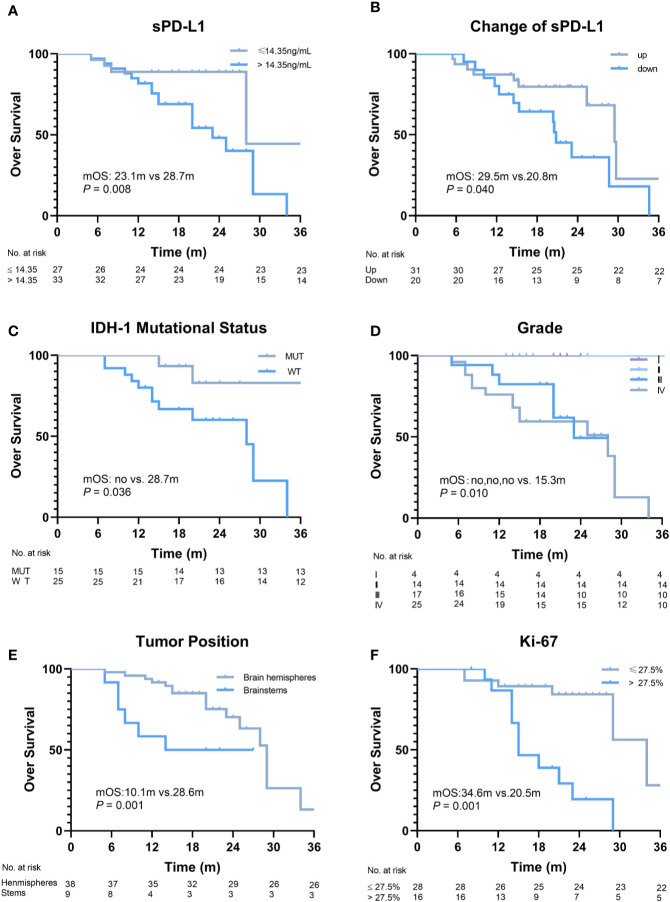
Overall survival (OS) of patients according to different factors. **(A)** The baseline level of soluble PD-L1 1 (sPD-L1) (≤14.35 ng/ml *vs.* >14.35 ng/ml, *P* = 0.008). **(B)** Changes in sPD-L1 after radiotherapy (up *vs.* down, *P* = 0.04). **(C)** IDH-1 mutational status (mutation *vs.* wide type, *P* = 0.036). **(D)** Grade (I, II, III, *vs.* IV, *P* = 0.01). **(E)** Tumor position (brain hemispheres *vs.* brainstems, *P* = 0.001). **(F)** Ki-67 (≤27.5% vs. >27.5%, P = 0.001).

**Figure 3:**
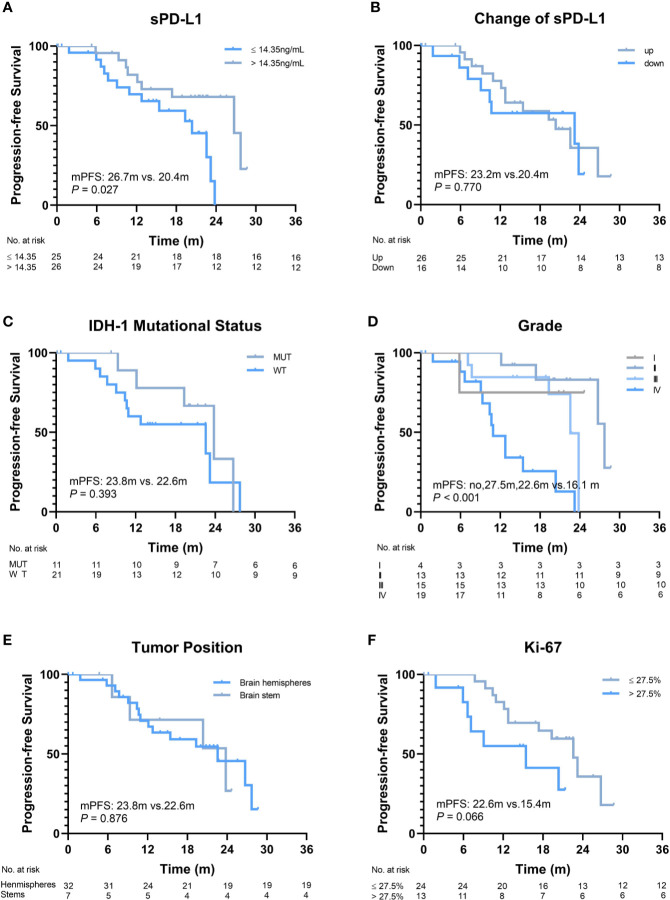
Progression-free survival (PFS) of patients according to different factors. **(A)** The baseline level of soluble programmed death-ligand 1 (sPD-L1) (≤14.35 ng/ml *vs.* >14.35 ng/ml, *P* = 0.027). **(B)** Changes in sPD-L1 after radiotherapy (up *vs.* down, *P* = 0.770). **(C)** IDH-1 mutational status (mutation *vs.* wide type, *P* = 0.393). **(D)** Grade (I, II, III *vs.* IV, *P* < 0.001). **(E)** Tumor position (brain hemispheres *vs.* brainstems, *P* = 0.876). **(F)** Ki-67 (≤27.5% vs. >27.5%, P = 0.066).

The median follow-up duration was 28.7 (range, 5.4–38.7) months. The disease of 23/60 (38.3%) patients progressed, and 22/60 (36.7%) patients died within the observation time. The median OS and progression-free survival (PFS) were 28.7 months and 23.2 months, respectively. To evaluate the predictive value of the baseline sPD-L1 level for survival, a cut-off value of 14.35 pg/ml was obtained using ROC curve analysis (AUC = 0.73; *P* = 0.003). Thirty-three patients (55%) had high sPD-L1 levels (> 14.35 pg/ml), and the other 27 patients had low sPD-L1 levels (≤ 14.35 pg/ml). Significantly worse median OS was noted in patients with higher baseline sPD-L1 levels than in those with lower baseline sPD-L1 levels (23.1 *vs.* 28.7 months, *P* = 0.008; **Figure 2A**). Additionally, patients with decreased sPD-L1 after RT had significantly worse median OS (20.8 *vs.* 29.5 months, *P* = 0.040) (**Figure 2B**). Other factors, including IDH-1 WT tumors (*P* = 0.036), GBMs (*P* = 0.010), tumors located in the brainstem (*P* = 0.001) and tumors with a Ki-67 expression rate >27.5% (*P* = 0.001), can also affect the OS of patients (**Figures 2C–F**). In this study, changes in sPD-L1 levels, IDH-1 mutational status and tumor location were independent prognostic factors (*P* = 0.003, HR = 0.019, 95% CI: 0.001–0.268; *P* = 0.011, HR = 0.029, 95% CI: 0.002–0.448; *P* = 0.002, HR = 26.302, 95% CI: 3.239–213.550) (**Table 2**). However, the baseline sPD-L1 level was not an independent prognostic factor for glioma patients (*P* = 0.516, HR = 2.231, 95% CI: 0.198–25.126). For PFS, patients with sPD-L1 concentrations above 14.35 pg/ml had worse PFS than those with low sPD-L1 levels (20.4 *vs.* 26.7 months, *P* = 0.027; **Figure 3A**), but an sPD-L1 concentration >14.35 pg/ml was not proven to be an independent prognostic factor for glioma patients in the multivariate analysis. High tumor grade was a poor prognostic factor for PFS both in univariate analysis (*P* < 0.001) (**Figure 3D**) and in multivariate analysis (*P* = 0.001, HR = 3.091, 95% CI: 1.592–6.002) (**Table 2**). As for other factors, including the change of sPD-L1, IDH-1 mutational status, tumor position, and Ki-67 expression, none of them exhibits a significant correlation with the PFS of glioma patients (**Figures 3B, C, E, F**).

**Table 2:** Univariable and multivariable analyses of OS and PFS in the patients.

Parameter	OS				PFS			
	Univariate analysis	Multivariate analysis			Univariate analysis	Multivariate analysis		
	*P*	*P*	HR	95%CI	*P*	*P*	HR	95%CI
Gender (male vs. female)	0.642				0.656			
Age (≤52 years vs. >52 years)	0.783				0.952			
Grade (I, II, III vs. IV)	0.010*	0.855	1.158	0.242–5.534	<0.001*	0.001*	3.091	1.592–6.002
IDH-1 (WT vs. MUT)	0.036*	0.011*	0.029	0.002–0.448	0.393			
sPD-L1 (≤14.35 pg/ml vs. >14.35 pg/ml)	0.008*	0.516	2.231	0.198–25.126	0.027*	0.617	0.737	0.223–2.435
Ki-67 (≤27.5% vs. >27.5%)	0.001*	0.846	0.824	0.117–5.818	0.066	0.635	1.278	0.464–3.524
Position (brain hemispheres vs. brainstem)	0.001*	0.002*	26.302	3.239–213.550	0.876			
Treatment (RT vs. RT + chemotherapy ± target	0.708				0.715			
Change in sPD-L1 (up vs. down)	0.040*	0.003*	0.019	0.001–0.268	0.770			

Factors with P ≤ 0.1 in univariate analysis can be included in the multivariate analysis.

OS, overall survival; PFS, progression-free survival; HR, hazard ratio; CI, confidence interval.

*P ≤ 0.05.

### Radiation Increases sPD-L1 Levels in Glioma Patients

**Figure 4:**
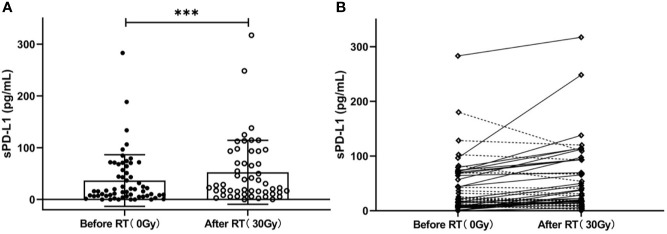
Changes in the soluble PD-L1 (sPD-L1) levels after radiotherapy (RT). **(A)** Overall change in sPD-L1 levels in patients (before RT *vs.* after RT: 36.65 ± 49.77 pg/ml *vs.*57.21 ± 60.95 pg/ml, *P* < 0.001). **(B)** Individual change in sPD-L1 levels (increased in 31 patients and reduced in 20 patients). ***P < 0.001. Data are presented as mean ± SD.

To explore whether radiation can induce sPD-L1 accumulation, we also measured the sPD-L1 levels in plasma samples of 51 out of 60 patients after RT (30 ± 10 Gy, 2 Gy/f), and found that their mean sPD-L1 levels after RT were significantly higher than the baseline sPD-L1 levels before RT (57.21 ± 60.95 pg/ml *vs.* 36.65 ± 49.77 pg/ml, *P* < 0.001) (**Figure 4A**). In details, sPD-L1 levels were increased in 31 patients and were reduced in 20 patients (**Figure 4B**).

Next, we undertook further analysis to measure other potential factors that might influence sPD-L1 levels. We first assessed the IDH-I mutational status by pathology analysis. In IDH-1 MUT group, the mean sPD-L1 levels were 17.52 ± 25.50 pg/ml before RT and 36.60 ± 39.66 pg/ml after RT, while they were 66.40 ± 66.55 pg/ml before RT and 70.32 ± 68.96 pg/ml after RT in the IDH-1 WT group. The baseline sPD-L1 level was significantly higher in the IDH-1 WT group than in the IDH-1 MUT group (*P* = 0.016); however, there was no statistical significance after RT between the two groups (*P* = 0.107) (**Figure 1B**). These results showed that sPD-L1 levels tended to increase in these two groups after RT, whereas the fold-change in the IDH-1 MUT group seemed more prominent. Conversely, in the treatment scheme subgroup analysis, the results showed that there were no significant differences in sPD-L1 levels among the RT, CRT, and CRT+T groups either before or after treatment (before treatment: 41.17 ± 43.85 pg/ml, 27.04 ± 30.96 pg/ml and 50.73 ± 81.71 pg/ml, respectively, *P* = 0.332; after treatment: 56.69 ± 47.52 pg/ml, 42.18 ± 53.46 pg/ml, and 71.56 ± 95.54 pg/ml, *P* = 0.432. **Figure 1D**). Thus, the addition of chemotherapy and bevacizumab to RT did not further influence the levels of sPD-L1 in this study.

### Anti-PD-L1 Antibody Could Reduce the Expression of sPD-L1 in Glioma Murine Model

**Figure 5:**
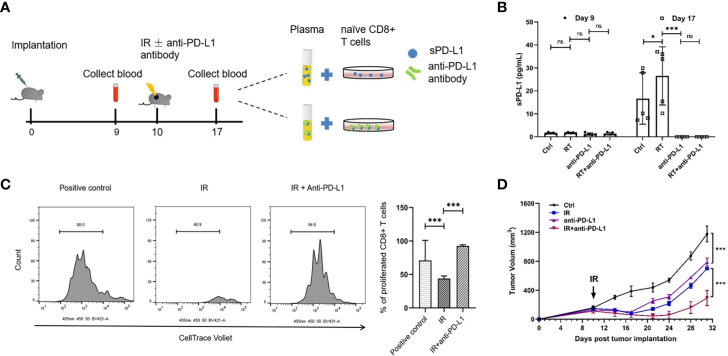
Anti-PD-L1 antibody could reduce the expression of sPD-L1 in glioma murine model. The glioma murine models were divided into ionizing radiation (IR) (20 Gy), anti-PD-L1, IR plus anti-PD-L1, and control groups (n = 5). **(A)** The scheme of *in vivo* experiments using glioma murine model. **(B)** The soluble PD-L1 (sPD-L1) were measured before and after IR, respectively, there was no difference in baseline expression levels in different groups; however, IR can upregulate the expression of sPD-L1 and anti-PD-L1 can effectively reduce the expression of sPD-L1. **(C)** Naive CD8+ T cells were cocultured with the plasma of mice after IR and subjected to suppression assay. Ratio indicates proliferated CD8+ T cells. It showed that suppression on T-cell proliferation can be enhanced upon sPD-L1 incubation *in vitro*. **(D)** Treated tumor was measured every 3–4 days for 21 days starting from the day of IR. The combination of IR and anti-PD-L1 could reduce tumor growth than either monotherapy. ns, nonsignificant. *P < 0.05; ***P < 0.001. Each experiment was repeated three times. Data are presented as mean ± SD.

To address whether circulating sPD-L1 molecules are directly targeted by an anti-PD-L1 antibody, we performed *in vivo* studies using the glioma murine model treated with IR (20 Gy), anti-PD-L1 or IR plus anti-PD-L1; we found that there was no difference in baseline sPD-L1 expression levels among the different groups (1.58 ± 0.315 pg/ml, 1.69 ± 0.24 pg/ml, and 1.18 ± 0.51 pg/ml, respectively, *P* = 0.227; **Figures 5A, B**). In line with the clinical data, an increase in the expression of sPD-L1 was observed after IR compared with the expression levels in the nonirradiated control group (16.68 ± 11.22 pg/ml *vs.* 28.50 ± 11.18 pg/ml, *P* = 0.031, **Figure 5B**). Notably, the concentration of sPD-L1 could not be detected in either the anti-PD-L1 alone group or the IR plus anti-PD-L1 group (**Figure 5B**), suggesting that sPD-L1 can be blocked by PD-L1 antibody, which can lead to significant downregulation of sPD-L1.

Since CD8^+^ T cells is crucial for radiation-induced anti-tumor effects, we sought to examine whether sPD-L1 affects the CD8^+^ T cells activation in the adaptive immune response. We performed the CD8^+^ T cells suppression analysis using sPD-L1-included mice plasma (**Figure 5A**). The plasma from the mice without anti-PD-L1 treatment group exhibited the remarkable CD8^+^ T cell suppression capacity (**Figure 5C**). The plasma from mice after anti-PD-L1 group (the sPD-L1 concentration is undetectable) didn’t show any suppression activity (**Figure 5C**). These indicate that the sPD-L1 plays an important role in T cell suppression in tumors.

We next sought to validate whether targeting sPD-L1 using anti-PD-L1 antibody can be a potential cancer therapy. We monitored the tumor size in glioma-bearing mice with different treatments. The IR or anti-PD-L1 antibody alone can slow down tumor growth (**Figure 5D**). Notably, the combination of IR and anti-PD-L1 significantly reduced tumor growth than either monotherapy (anti-PD-L1 *vs.* IR plus anti-PD-L1: 789.67 ± 55.86 mm^3^ *vs.* 292.16 ± 102.98 mm^3^ on day 31, *P* < 0.001; IR *vs.* IR plus anti-PD-L1 = 697.02 ± 12.98 mm^3^ *vs.* 292.16 ± 102.98 mm^3^ on day 31, *P* < 0.001) (**Figure 5D**).

## Discussion

Circulating sPD-L1 in the blood has recently been discovered in various malignancies. However, few studies have reported sPD-L1 expression in patients with glioma until now (21). To further explore the existence of sPD-L1 and evaluate the pathological significance of this circulating factor in human cancer, we developed this study for the detection and quantification of sPD-L1 in glioma patients receiving RT. In the present study, by using ELISA formats, we detected that approximately 90% of glioma patients expressed sPD-L1 in the plasma before RT. Further, we determined that RT could upregulate sPD-L1 levels compared with baseline levels. In addition, the high baseline level of sPD-L1, decreased level of sPD-L1 after RT and some other clinical factors, such as IDH-1 WT tumors, GBMs, tumors located in the brainstem and tumors with a Ki-67 expression rate > 27.5%, were demonstrated to be related to poor prognosis in glioma patients. Using the glioma murine model, our data showed that anti-PD-L1 antibody combined with radiation can be an effective therapy method.

The PD-L1/PD-L1 axis is associated with the tumor microenvironment as a regulator of inhibitory signals, and its expression could be a candidate biomarker for patient selection for anti-PD-L1/PD-L1 monoclonal therapy. Aberrant PD-L1 expression has already been reported to occur in glioma tumor tissues based on IHC (8, 23). Considering that the sPD-L1 level may be associated with the tumor burden and the aggressive biological activities of tumors, we investigated whether there were associations between the baseline level of sPD-L1 and the tumor grade or Ki-67 expression, and finally demonstrated that the baseline level of sPD-L1 was significantly elevated in patients with advanced brain tumors or in patients with Ki-67 > 27.5%. Ki-67 is one of the most widely used markers for proliferation in clinical practice and has been validated as a marker in the initial phase of adult neurogenesis (24). Although the mechanism remains unclear, we speculated that most sPD-L1 may be shed from the surface of cells in tumors by the cleavage of membrane-bound proteins and is found in free form in the plasma. In addition, circulating sPD-L1 could lead to immune tolerance; consequently, neoplastic cells would have no limits to proliferation. Therefore, sPD-L1 may be considered to exist from the early stage of glioma progression. In recent years, distinct molecular classes of gliomas have been identified. It is well established that IDH-1 MUT and IDH-1 WT gliomas have distinct tumor behavior driven by different oncogenic signals and respond differently to current treatment paradigms. Notably, by comparing the immune responses between IDH-1 MUT and IDH-1 WT patients, some studies have identified a marked reduction in the expression of immune-related genes, including the *CD274* (PD-L1 coding gene) gene, in IDH-1 MUT gliomas (25–27). The results were in line with ours, and downregulated sPD-L1 levels tended to occur in glioma patients with IDH-1 MUT tumors. Collectively, these findings may suggest that the immunological tumor microenvironment may differ according to the IDH mutational status of gliomas.

In addition to the association with some clinical factors, sPD-L1 levels might predict the survival outcomes in cancer patients; however, its prognostic relevance was contradictory in different cancers. In gastric adenocarcinoma, elevated levels of sPD-L1 were associated with a favorable prognosis (65.6% *vs.* 44.7%, *P* = 0.028) (28). Inversely, studies in natural killer/T-cell lymphoma (NKTCL), aggressive renal cell carcinoma (RCC), NSCLC, large B-cell lymphoma, HCC, and nasopharynx cancer (NPC) demonstrated that patients with high concentrations of sPD-L1 exhibited markedly worse survival than patients with lower concentrations (18–20, 29, 30). In the current study, we observed that high baseline levels of sPD-L1 (>14.35 pg/ml) in glioma patients were correlated not only with poorer OS (23.1 *vs.* 28.7 months, *P* = 0.008) but also with poorer PFS (20.4 *vs.* 26.7 months, *P* = 0.027) in univariate analysis. The biological reason why sPD-L1 is more strongly associated with survival outcomes has to be further elucidated. It is very possible that as sPD-L1 spreads throughout the body *via* the blood and lymphatic circulation, it exerts a widespread inhibitory effect on T cells by interacting with cell surface receptors such as membrane-bound PD-1 (31–33). And this hypothesis was verified by T cell suppression assay in this study. In addition, we found that sPD-L1 molecules might represent a direct target of therapeutic PD-L1 antibodies. The described functions might work as mechanisms of escape from immune surveillance and/or result in an impairment of anti-PD-1/PD-L1-directed antibody therapy and thus translate into prognostic and/or predictive factors in cancer patients. Altogether, the quantification of circulating sPD-L1 may also be of use as a predictive marker of anti-PD-1 treatment outcome, helping clinicians select patients for PD-1/PD-L1-based therapy strategies. Thus, further studies with a large number of patients are required to clarify our findings.

Next, we attempted to uncover the dynamics of circulating sPD-L1 levels in glioma patients undergoing RT. It is known that RT complicates the interpretation of the immune landscape in patients. The ultimate goal of the combination of immunotherapy and RT is to achieve a long-lasting, therapy-induced immune response at all sites of disease, and the assessment of the dynamic changes in the immune system at the patient level is essential. Our group analyzed the changes in the sPD-L1 level before and following RT and found that RT significantly increased sPD-L1 expression in both patients with glioma and glioma murine model. Similarly, Hyun et al. reported that RT significantly increased sPD-L1 expression in patients with HCC (34). However, most investigations have focused only on the PD-L1 level at baseline, and data on changes in PD-L1 expression after RT are still extremely limited.

In the sub-analysis of this study, we also noticed that sPD-L1 levels were increased significantly in IDH-1 MUT patients compared with IDH-1 WT patients. To the best of our knowledge, this is the first prospective study to evaluate the sPD-L1 levels following RT in glioma patients with different IDH mutational statuses. Our finding can be explained by the fact that IDH-1 mutation could apparently improve the sensitivity of tumors to RT, and then cells in tumors killed by RT could release an abundance of sPD-L1 into the blood. However, we also found that the addition of chemotherapy or/and bevacizumab to RT did not further upregulate the increase in sPD-L1 compared with RT only. We suspect that RT as a local therapy provides sufficient damage to the tumor target, and there was probably no room to further improve treatment efficacy with the addition of chemotherapy and/or bevacizumab beyond that of RT in the primary tumor. Taken together, the sPD-L1 level increased after RT, suggesting that RT combined with immune checkpoint inhibitors might be better than RT alone for glioma patients, especially for patients with IDH-1 MUT. Further well-designed studies are needed to clarify the optimal RT scheme, dose, and time for combination.

Regarding the limitations of this study, first, this study had a limited sample size. Second, the only RT dose used was 30 ± 10 Gy; hence, we could not determine whether the sPD-L1 level will change after a larger dose of RT. Third, some patients underwent partial excision, and others underwent only biopsy, which might affect the sPD-L1 level.

## Conclusion

In conclusion, this study reported that sPD-L1 can be assayed in the plasma of glioma patients. This finding may mean that compensation for the potential sequestration of antibodies needs to be considered in the optimization of PD-L1 blockade therapies. Not all administered anti-PD-L1 immunotherapeutic antibodies may reach the surface of tumor cells, with a potentially appreciable proportion being sequestered by sPD-L1 within the circulation. In addition, the baseline level of sPD-L1 might be a potential marker to predict the outcome in glioma patients, which would truly be remarkable, because predictive biomarkers that discriminate responders from non-responders at therapy initiation are scarce. Finally, the elevated level of sPD-L1 after RT suggests that the combination of RT with immune checkpoint inhibitors may be a promising therapeutic strategy in gliomas, especially for patients with IDH-1 MUT gliomas.

## Data Availability Statement

The original contributions presented in the study are included in the article/supplementary materials. Further inquiries can be directed to the corresponding authors.

## Ethics Statement

The studies involving human participants were reviewed and approved by The Ethics Committee of the Shandong Cancer Hospital. The patients/participants provided their written informed consent to participate in this study. The animal study was reviewed and approved by The Ethics Committee of the Shandong Cancer Hospital.

## Author Contributions

X-CD was a major contributor in writing the manuscript. L-LW and Y-FZ analyzed and interpreted the patient and animal data. X-CD and JY did the experiments in the manuscript. J-MY, HL, RRW and MH designed the work and provided the financial support. S-LN and Y-DL revised the manuscript. All authors contributed to the article and approved the submitted version.

## Funding

This work was supported by National Key Research and Development Program of China (Grant No. 2018YFE0114100), Key Research and Development Program of Shandong province, China (Grant No. 2019GGX101057), Science Technology Program of Jinan (Grant No. 201805051) and The Innovation Project of Shandong Academy of Medical Sciences (2019-04), and the Academic Promotion Program of Shandong First Medical University (Grant No. 2019ZL002).

## Conflict of Interest

The authors declare that the research was conducted in the absence of any commercial or financial relationships that could be construed as a potential conflict of interest.
